# Henipavirus and Tioman Virus Antibodies in Pteropodid Bats, Madagascar

**DOI:** 10.3201/eid1301.060791

**Published:** 2007-01

**Authors:** Catherine Iehlé, Girard Razafitrimo, Josette Razainirina, Nicole, Andriaholinirina, Steven M. Goodman, Caroline Faure, Marie-Claude Georges-Courbot, Dominique Rousset, Jean-Marc Reynes

**Affiliations:** * Institut Pasteur de Madagascar, Antananarivo, Madagascar; † Field Museum of Natural History, Chicago, Illinois, USA; ‡ World Wide Fund for Nature, Antananarivo, Madagascar; § Institut Pasteur, Paris, France; ¶ Centre Pasteur du Cameroun, Yaoundé, Cameroon

**Keywords:** Madagascar, bats, henipavirus, Tioman virus, dispatch

## Abstract

Specimens were obtained from the 3 Malagasy fruit bats, *Pteropus rufus*, *Eidolon dupreanum*, and *Rousettus madagascariensis*. Antibodies against Nipah, Hendra, and Tioman viruses were detected by immunoassay in 23 and by serum neutralization tests in 3 of 427 serum samples, which suggests that related viruses have circulated in Madagascar.

The Old World fruit bats of the family *Pteropodidae*, particularly species belonging to the genus *Pteropus*, have been considered natural hosts for viruses emerging in Australia (Hendra virus [HeV], Australian bat lyssavirus [ABLV], and Menangle virus), Malaysia, Singapore, and Bangladesh (Nipah virus [NiV]) (*1*,*2*). The geographic distribution of the henipaviruses NiV and HeV or related unrecognized viruses may overlap with that of *Pteropus* spp. outside Australia, Malaysia, and Bangladesh. This hypothesis was confirmed with the evidence of NiV in *P*. *lylei* from Cambodia and Thailand (*3*,*4*). NiV emergence represents a human and animal health problem because the virus causes severe febrile encephalitis associated with death in humans and respiratory illness in domestic pigs. Isolated human cases have been reported for the 3 Australian viruses (*1*). Another paramyxovirus, the rubulavirus Tioman (TiV), was isolated from the urine of a *P*. *hypomelanus* bat, collected on Tioman Island, Malaysia (*5*). Although closely related to Menangle virus and associated with *Pteropus* spp., TiV has not yet been associated with any human disease.

Pteropodids have less species diversity in Madagascar than in other Old World tropical regions. Three species, all endemic, are found on the island: *P. rufus*, *Eidolon dupreanum*, and *Rousettus madagascariensis*. These species are distributed across much of the island and are more common in lowland areas than in the highlands (*6*). We report on a survey of Malagasy fruit bats to assess the presence and distribution of Hendra, Nipah, and Tioman (like) viruses on this island.

## The Study

The samples were collected May 2003–July 2005 at different locations (Figure), most with multiple sampling sites. Most fruit bats were captured during the dry season by using mist nets set near roosting places (trees or caves). Bats were immobilized face up, and a blood sample was taken using sterile procedures. Urine was collected directly from the bat’s urogenital opening, before the blood sampling, with a cotton swab that was immediately placed in a cryotube containing 1 mL viral transport medium. When a urine sample could not be obtained from a given animal, a pharyngeal sample was collected with a cotton swab that was then directly placed in the viral transport medium. Most animals were subsequently released. Urine samples were also collected under 1 *P*. *rufus* tree roost, as described previously (*7*).

**Figure:**
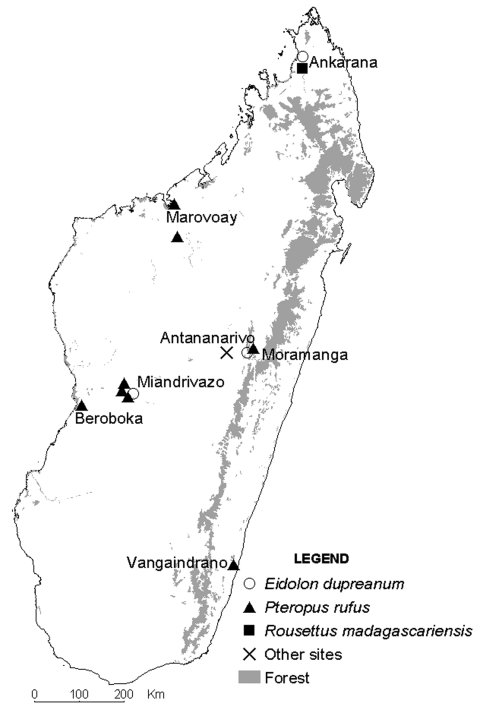
Madagascar, showing bat collection sites by species. For reference, the X indicates the location of the capital city, Antananarivo, where no samples were collected.

Blood was centrifuged in the field at ambient temperature at 3,000 rpm for 15 min. Serum, blood pellets, urine, and pharyngeal samples were placed in a container of charged nitrogen until their arrival at the laboratory, where they were stored at –80°C.

Bat sera were screened for antibodies against NiV, HeV, and TiV by enzyme-linked immunosorbent assay (ELISA), as described previously (*3*). In total, 427 serum specimens were tested (Table); 23 (5.4%) were positive for 1 of the viruses. Antibodies to NiV and HeV were mainly detected in *E. dupreanum* sera (14/73) but also in *P. rufus* sera (8/349), whereas antibodies to TiV were found in 2 *P*. *rufus* serum specimens and 1 *R. madagascariensis* specimen. All samples positive for antibodies to HeV were also positive for antibodies to NiV. ELISA-seropositive bats were detected in 5 of 7 locations investigated (Table; Figure).

**Table:** Serum samples ELISA-reactive to Nipah, Hendra, and Tioman viruses collected from fruit bats, Madagascar, 2003–2005

Species and location	No. tested	Seropositive samples			
		Nipah virus	Hendra virus	Tioman virus	Total (%)
*Eidolon dupreanum*					14/73 (19.2)
Marozevo (Moramanga)	53	10	10	0	10
Miandrivazo	2	0	0	0	0
Ankarana	18	4	1	0	4
*Pteropus rufus*					8/349 (2.3)
Marovitsika (Moramanga)	33	2	2	0	2
Miandrivazo	112	4	0	1	5
Marovoay	140	0	0	1	1
Beroboka	27	0	0	0	0
Vangaindrano	37	0	0	0	0
*Rousettus madagascariensis*					1/5 (20.0)
Ankarana	5	0	0	1	1
Total	427	20	13	3	23/427 (5.4)

Serum neutralization tests were carried out by using NiV, HeV, or TiV. Serum samples were heated for 30 min at 56°C and then titrated with 2-fold dilutions (1:10 to 1:640) as previously described (*3*). Positive control for the NiV and HeV tests was an anti-NiV serum sample obtained from a convalescent-phase patient. Positive control for the TiV test was serum collected from a hamster experimentally infected with TiV. Of the 23 ELISA-positive serum samples, 21 could be tested by neutralization test, using NiV, HeV, and TiV. Neutralizing antibodies to NiV and HeV were detected in 2 of the 13 *E*. *dupreanum* serum samples that were ELISA positive for HeV, NiV, or both (titers 1:40 and 1:80 for NiV, 1:20 and 1:10 for HeV, respectively). Another *E*. *dupreanum* bat was also confirmed positive only for antibodies to NiV (1:40); the 10 other *Eidolon* bats were negative. One bat that was ELISA seropositive for NiV and HeV *P*. *rufus* was confirmed positive for neutralizing antibodies to HeV (1:160) and was found to be positive for neutralizing antibodies to TiV (1:80); the 7 other *Pteropus* bats were negative. All ELISA-positive sera confirmed by the neutralization test were obtained in the same geographic area, Moramanga District (Figure).

Virus isolation experiments were performed on 118 urine and 285 pharyngeal specimens. Thus, subconfluent Vero E6 cells (ATCC CRL-1586) were inoculated with 500 μL of viral transport medium containing a cotton swab impregnated with urine or pharyngeal epithelial cells, as previously described (*3*). Results were negative, but 22 were inconclusive because of bacterial and fungal contamination.

## Conclusion

Our study provides the first evidence that the 3 pteropodid bats on Madagascar (*P. rufus*, *E. dupreanum*, and *R. madagascariensis*) have been in contact with viruses of the *Paramyxoviridae* family, and especially of the genus *Henipavirus*. The distribution of *Pteropus* is limited to islands of the Pacific and Indian Oceans and continental areas from Pakistan east across Southeast Asia to Australasia. The genus *Rousettus* is found in both Africa and Asia; the genus *Eidolon,* only in Africa and Madagascar. The presence of antibodies to henipaviruses (detected by ELISA or serum neutralization test) in *Eidolon* could suggest the possible presence of these viruses in Africa, where the only other species in this genus, *E*. *helvum*, is found. The distributional pattern of *Eidolon* indicates that a dispersal event occurred between Africa and Madagascar (*8*). A lateral transfer on Madagascar between *E*. *dupreanum* and the other pteropodids could have occurred on that island. *E*. *dupreanum* and *R*. *madagascariensis* are known to share cave roost sites during the day (*9*) and, with *P. rufus*, can be found feeding at night in the same fruit trees (*10*). A survey for henipaviruses in Africa should be considered to confirm the hypothesis of a wider distribution of these pathogens.

In Cambodia, the circulating NiV isolated strain was very similar to the Malaysian strain used in ELISA and serum neutralization tests. Therefore, most of the ELISA-positive bat serum specimens (95%) were confirmed by the serum neutralization test (*3*). In our Madagascar study, in which the Malaysian strain was also used, few ELISA-positive specimens (16%, n = 19) could be confirmed by the serum neutralization test. We hypothesize that henipaviruses circulate in Madagascar and are sufficiently divergent from the NiV and HeV strains used in this study, which explains these discordant results. To attempt to isolate and characterize the circulating viruses, a long-term survey among pteropodids is being conducted in the Moramanga District, where positive neutralization test serum specimens were obtained.

Day roost sites of Malagasy pteropodids are extremely rare in proximity to human settlements. However, these animals are hunted for food and can be found alive or dead in local markets and in restaurants. Further, humans eat fruits from trees where pteropodids have fed. More research is needed to clarify the possible risk of pathogen transmission to humans.
